# Why the Central Dogma: on the nature of the great biological exclusion principle

**DOI:** 10.1186/s13062-015-0084-3

**Published:** 2015-09-16

**Authors:** Eugene V. Koonin

**Affiliations:** National Center for Biotechnology Information, National Library of Medicine, National Instittues of Health, Bethesda, MD 20894 USA

**Keywords:** Central Dogma, Digital information, Analogous information, Translation, Aminoacyl-tRNA synthetases

## Abstract

**Abstract:**

The Central Dogma of molecular biology posits that transfer of information from proteins back to nucleic acids does not occur in biological systems. I argue that the impossibility of reverse translation is indeed a major, physical exclusion principle that emerges due to the transition from the digital information carriers, nucleic acids, to analog information carriers, proteins, which involves irreversible suppression of the digital information.

**Reviewers:**

This article was reviewed by Itai Yanai, Martin Lercher and Frank Eisenhaber.

## Background

**Fig. 1:**
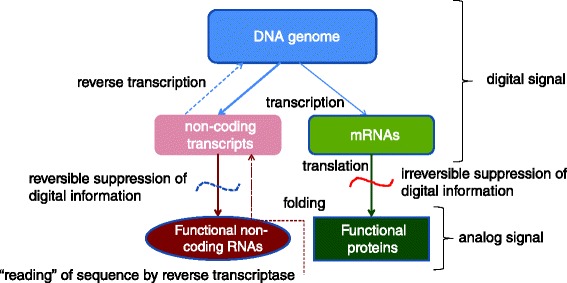
Digital and analog information carriers and transfer routes in biological systems

To a large extent, physics centers around major exclusion principles (constraints) which indicate which kinds of processes are prohibited by the laws of nature. Such is the nature of the laws of thermodynamics as well as the Pauli exclusion principle in quantum physics [1]. Obviously, these laws apply to the biological realm but as far as specific biological exclusion principles go, there seems to be only one: the so-called Central Dogma of Molecular Biology [2, 3]. All cellular life forms share the same fundamental scheme of genome replication and expression that has been formulated by Francis Crick in the classic 1970 article that was inspired by the discovery of the reverse transcriptase (RT) (Fig. 1) [2]. Crick presciently noted that there was only one truly fundamental principle at the heart of the Central Dogma: there is no route of reverse information transfer from proteins to nucleic acids, i.e. no reverse translation. In contrast, all the transitions between different forms of nucleic acids are, in principle, permitted and occur on some occasions.

In more abstract terms, the unique, unidirectional route of information transfer represents the shift from digital to analogous encoding of information, in other words, the transition between the fundamentally one-dimensional (digital) information contained in nucleic acids to the three-dimensional, analog form of information embodied in proteins [4]. The reverse process of recoding from analog to digital information is prohibited according to the Central Dogma. Why should this be the case? The question does not appear trivial or moot. Certainly, reverse translation would require an elaborate molecular machinery but conceivably, it would not have been more complex than the translation system. In principle, one could imagine a “Lamarckian evolution machine” whereby protein sequence would change in response to environmental cues and then such protein mutations would be then imprinted in the genome. All evolution of life would have been radically different from the version realized on earth but it is not a priori obvious that this type of life is untenable. Are there fundamental reasons why this route of information transmission has not evolved? I discuss such possible reasons below.

## Irreversibility of the digital to analog transition

The key step of the recoding from the digital to the analog representation of information involves the specific aminoacylation of cognate tRNAs catalyzed by the aminoacyl-tRNA synthetases (aaRS). The aaRS recognize, on the one hand, individual amino acids, which they activate via conjugation with AMP, and on the other hand, the cognate tRNA molecules to which the aaRS transfer the amino acid residues [5]. Thus, the aaRS join, through the tRNA molecule, an element of the analog signal (an amino acid) with its digital cognate, the tRNA anticodon and accordingly are responsible for the information transition. At the next step of translation, the ribosomes, intricate molecular machines as they are, only exploit digital information through codon-anticodon pairing.

Translation is accompanied or followed by protein folding, and this process involves “forgetting”, or perhaps more precisely, irreversible suppression of the digital information (Fig. 1). When a protein folds into its native conformation, amino acid residues that are distant in the sequence (one dimension) become juxtaposed in three dimensions such that the sequence cannot be accurately “read” without denaturing the protein. When the native conformation of a protein is disrupted, the outcome is a misfolded globule not an extended one-dimensional string [6]. The existence of such a string is thermodynamically untenable, hence the irreversibility of the suppression of the digital message. Misfolded proteins can be toxic for cells and are swiftly destroyed by the elaborate protein degradation machinery [7]. The case of intrinsically disordered proteins appears somewhat different but few proteins are fully disordered [8]. Thus, the irreversible suppression of the digital information engendered by the formation of the analog device, i.e. protein folding, appears to be the fundamental cause of the irreversibility of translation, that is, the Central Dogma.

## Evolution of the digital to analog transition

Proteins are not the only form of analog devices in biological systems. Some key function including those in translation are performed by non-coding RNAs, and the repertoire of non-coding RNA molecules with known biological roles is rapidly expanding [9]. The non-coding RNA molecules function in three dimensions and in that sense are analog devices. Remarkably, however, the digital properties of the RNA molecules are only reversibly suppressed. The RNA sequence can be unmasked and “read” by reverse transcriptase (or in some cases, RNA-dependent RNA polymerase) resulting in genomic imprinting of changes that accumulate in RNA molecules. Thus, functional RNA molecules are hybrid devices that fully retain their digital properties and their formation is thus not associated with any exclusion principles.

The leading hypothesis on the early stages of the evolution of life is the RNA World, a hypothetical form of biology where both the role of information carriers and the function of operational devices belong to RNA molecules [10, 11]. The RNA World has not been reproduced in the laboratory, and success of such experiments is difficult to predict for the foreseeable future. Nevertheless, this evolutionary scenario appears extremely plausible both on the force of arguments stemming from comparative genomics [12] on the possible course of evolution and owing to the growing number of catalytic activities shown to be attainable with RNA molecules (ribozymes) [13]. The RNA World would have been a form of life that was fully permeated by digital information albeit with a substantial analog component. This form of biology would not exclude any routes of information transmission that were accessible within the available chemistry.

Why then the advent of the more complex analog devices, the proteins, the irreversible suppression of digital information and the ensuing Central Dogma? The simple, more or less obvious answer is that the exclusion principle is the cost to pay for the dramatically greater molecular versatility of proteins compared to RNA. Apparently, only limited complexity could have been reached by using RNA molecules as operational devices. To achieve the cellular level of complexity, the substrate employed for the digital information carriers (nucleotides) had to be abandoned for more diverse building blocks (amino acids) and the digital nature of information in proteins had to be suppressed through the distinct process of polypeptide chain folding.

A wondrous aspect of biological evolution seems to be that all mechanisms that are physically feasible appear to be realized in some systems, even if on rare occasions. Thus, analog replication, although not central to biology, does occur in the course of prion propagation [14]. Furthermore, the route of information transfer from protein to the genome might not be completely blocked (so formally, the strict validity of the Central Dogma could be questioned [15]). However, if such transfer does indeed occur, the mechanism is by no account a reversion of the translation process. Rather, it appears to involve the so-called assimilation process whereby somatic mutations, such as amino acid misincorporation in proteins, that exert a particular phenotypic effect are occasionally recapitulated by genetic mutations [15]. In contrast, the “ban” on reverse translation seems to be a physical exclusion principle that emerges along the route of transition from the digital to analog information carriers.

## Conclusions

The Central Dogma of molecular biology essentially captures the irreversibility of the translation process. This irreversible character of translation seems to reflect a major exclusion principle that emerges due to the irreversible suppression of digital information along the path of transition from digital information carriers, nucleic acids, to analog information carriers, proteins, in the course of translation.

## Reviewers’ reports

### Reviewer 1: Itai Yanai, The Technion, Haifa, Israel

In this short manuscript, Koonin argues that the most important aspect of the Central Dogma is that it forbids reverse translation. Koonin has previously written in this journal about the problem of evolving from an RNA-world to a nucleic-acid system coupled with a protein system, offering the anthropic principle as one solution. Here he notes that the transition was one from a digital information system to an analog one. This transition benefited from a wide range of variation thus leading to better fitness over time, however was also accompanied with the cost of broadly preventing a bidirectional flow of information. This historic compromise is now a central aspect of biology on Earth, and Koonin argues that for this reason the Central Dogma should indeed be considered central. I find this piece interesting and compelling; seeming obvious only because that is the hallmark of all good ideas once they are proposed. In particular, I am very happy to see Koonin take a concept that was developed by a molecular biologist and frame it in an evolutionary context. It urges me to think that other central biological concepts would benefit from such re-framing.

Authors’ response: These thoughtful, constructive comments are much appreciated.

### Reviewer 2: Martin Lercher, University of Duesseldorf

The “Central Dogma” of molecular biology hypothesizes the impossibility of converting the amino acid sequence of a protein back into a nucleic acid sequence. (Nota bene: I cringe having to write “Dogma” in a science context, but the misnomer has stuck.) In his paper, Eugene Koonin convincingly argues that the exclusion of reverse translation is due to the fact that the “analog” 3-D structure of proteins cannot be reverted back to a linear amino acid sequence. Thus, “digital” (=linear sequence) information is lost in protein folding and cannot be recovered. In retrospect, it seems surprising that this issue has not received more attention previously–the hallmark of original thinking.

Authors’ response: This is an excellent way to summarize the gist of the paper, and I truly appreciate it.

I have only two comments. First, the general sequence of transitions is conceptually identical between functional RNA and amino acid sequences: (1) a linear sequence in a fixed molecular alphabet = primary structure (termed “digital information” by Koonin) → (2) pairing between specific molecules = secondary structure → (3) folding into a 3-D structure = tertiary structure (termed “analog information” by Koonin) While cellular machineries are able to convert the 3-D structure (“analog information”) back to the corresponding linear sequence (“digital information”) for RNA, the same is apparently not true in the case of amino acids. It would be interesting to ponder why that is–is it because the molecular interactions between amino acids are orders of magnitudes stronger, or is it because these interactions are not always pairwise? To fully understand why reverse transcription is possible while reverse translation is not, it would be important to better understand the fundamental (?) difference between RNA tertiary structure and protein tertiary structure.

Authors’ response: This is indeed a fundamentally important issue that is partially addressed in the article. In particular, it is stated that “When the native conformation of a protein is disrupted, the outcome is a misfolded globule not an extended one-dimensional string.” This is mostly the case because protein folding in three dimensions is based, mostly, on distant rather than local interactions. In RNA folding, local interactions (hairpins) play a much greater role. Therefore, after being reverse-transcribed, the RNA easily enough folds back into the native information, thus avoiding the problems caused by accumulation of misfolded molecules, in a sharp contrast to the situation with proteins. Furthermore, and perhaps even more importantly, RNA folding is based primarily on the very same complementary interactions between bases as RNA synthesis. Accordingly, the “Crick Demon”, i.e. the reverse transcriptase, is relatively simple device. The “Anfinsen Demon”, the hypothetical machinery for reverse translation, would have to be incomparably more complex. Thus, there seem to be no thermodynamic reasons why the “Anfinsen Demon” could not exist but the biological reasons appear compelling.

Second, I found the terminology of “digital” and “analog” information somewhat confusing. The linear amino acid sequence (the “digital” information) is still present in the tertiary structure, and a Maxwell-like demon could walk along this sequence to report it (which is essentially what the reverse transcriptase does in the case of RNA). In principle, no information (except codon usage) is lost in the transitions between the different layers of structure. This is in contrast to, e.g., the encoding of music: the digital signal is irrevocably lost in the conversion to an analog signal, and repeated conversions between analog and digital will lead to increasing deviations in both signals. Thus, the digital/analog juxtaposition may be more an analogy than a precise description, and pointing that out would increase readability.

Author’s response: I have to agree, the digital vs analog opposition here is more of an analogy than a precise description. Indeed, an “Anfinsen Demon” could exist, in principle, not being prohibited by thermodynamics. However, as pointed out above, there are major biological reasons why it has never evolved: i) the actions of such a demon would wreak havoc to the cell, leaving behind misfolded proteins, unless an entire flock of demons was dedicated to refolding, ii) the demon would have to be extremely complex, at least as complex as the translation system. I believe that, given that in Biology Direct, the reviews and responses an integral part of the article, these comments will serve to clarify and increase readability.

I don’t see how thermodynamic laws are exclusion principles–they are approximations for the behavior of large populations.

Authors’ response: Here I have to respectfully disagree. Beyond doubt, the laws of thermodynamics are approximations for the behavior of large populations but that does not preclude them from being exclusion principles. Indeed, they expressly forbid the existence of perpetual motion machines of the first kind (the first law) and the second kind (the second law).

### Reviewer 3: Frank Eisenhaber (with Birgit Eisenhaber), Singapore Institute of Bioinformatics

This review is the result of joint effort by Birgit Eisenhaber and Frank Eisenhaber. The proposed MS about the central dogma of molecular biology provides a welcome re-consideration of the dogmatic presentation of the matter in textbooks. It was a pleasure to read the text and it sparked a ping-pong of arguments between us. First, it is a good idea to put the central dogma into one row with fundamental physical laws of exclusion and conservation and to look for the justification at the physical and not so much at the biological level. The second idea of seeing the problem in the digital-analogue transformation context is yet another intellectual breakthrough with the implied need for readout from fully denaturated protein chains. We wish to emphasize the additional thought that the way back from proteins to nucleic acids is also blocked by the problem of non-uniqueness (Nichteindeutigkeit), the disambiguation of many possible return paths. First, one AA is represented by several codons. They might not differ in their translation values but they affect expression fidelity at both transcription and translation. Should the cells learn how to measure relative expression or to amplify a gene with reflection of all observed changes (including the inclusion into appropriate expression frameworks)? Further, there might be several isoforms and also mutations at the same site in the same protein in the same cell. How to resolve this ambiguity, again by finding the more frequent mutant? Third, the protein experiences lots of PTMs in its life time including those who change the sequence itself. Latest, the connection to the original gene gets lost at the level of proteolytic maturation and AA identity changing chemistry.

Author’s response: This constructive and interesting review is greatly appreciated.

## Abbreviations

aaRS: Aminoacyl-tRNA synthetase
